# Genetic and other risk factors for suicidal ideation and the relationship with depression

**DOI:** 10.1017/S0033291717000940

**Published:** 2017-05-08

**Authors:** R. Dutta, H. A. Ball, S. H. Siribaddana, A. Sumathipala, S. Samaraweera, P. McGuffin, M. Hotopf

**Affiliations:** 1 Department of Psychological Medicine, Institute of Psychiatry, Psychology and Neurosciences, King's College London, London SE5 9RJ, UK; 2 Sri Lanka Twin Registry, Institute of Research and Development, Battaramulla, Sri Lanka; 3 Faculty of Medicine, Rajarata University of Sri Lanka, Saliyapura, Sri Lanka; 4 Keele University, Stoke-on-Trent, UK

**Keywords:** Depression, genetics, suicidal ideation, twin study

## Abstract

**Background:**

There is a genetic contribution to the risk of suicide, but sparse prior research on the genetics of suicidal ideation.

**Methods:**

Active and passive suicidal ideation were assessed in a Sri Lankan population-based twin registry (*n* = 3906 twins) and a matched non-twin sample (*n* = 2016). Logistic regression models were used to examine associations with socio-demographic factors, environmental exposures and psychiatric symptoms. The heritability of suicidal ideation was assessed using structural equation modelling.

**Results:**

The lifetime prevalence of any suicidal ideation was 13.0% (11.7–14.3%) for men; 21.8% (20.3–23.2%) for women, with no significant difference between twins and non-twins. Factors that predicted suicidal ideation included female gender, termination of marital relationship, low education level, urban residence, losing a parent whilst young, low standard of living and stressful life events in the preceding 12 months. Suicidal ideation was strongly associated with depression, but also with abnormal fatigue and alcohol and tobacco use. The best fitting structural equation model indicated a substantial contribution from genetic factors (57%; CI 47–66) and from non-shared environmental factors (43%; CI 34–53) in both men and women. In women this genetic component was largely mediated through depression, but in men there was a significant heritable component to suicidal ideation that was independent of depression.

**Conclusions:**

These are the first results to show a genetic contribution to suicidal ideation that is independent of depression outside of a high-income country. These phenomena may be generalizable, because previous research highlights similarities between the aetiology of mental disorders in Sri Lanka and higher-income countries.

## Introduction

The World Health Organisation (WHO) estimates that 75% of suicides occur in low and middle-income countries (LMICs), yet research from these areas is scarce (Hawton, 2014; WHO, 2014). Sri Lanka, the setting for this study, ranks fourth out of 172 countries (WHO, 2014) with a suicide rate of 28.8/100 000/year compared with a global rate of 11.4/100 000/year in 2012.

Suicidal ideation is a risk factor for completed suicide (O'Connor & Nock, 2014) and there are marked international differences in the prevalence. The SUPRE-MISS study (Bertolote *et al.* 2005) reported prevalence of suicidal ideation ranging from 2.6% in Chennai, India to 25.4% in Durban, South Africa.

Family studies in high-income countries suggest that completed suicide and suicide attempts may be familial and at least partly independent from the heritability of psychiatric disorders (Brent & Melhem, 2008).

There is contradictory evidence whether suicidal *ideation* is part of a familial phenotype for suicidal behaviour. Two large-scale adult epidemiological twin studies in Australia (Statham *et al.* 1998) and the USA (Fu *et al.* 2002) have suggested there is heritability for lifetime suicidal ideation as part of a suicidal spectrum phenotype (Schosser *et al.* 2011), whereas other studies suggest ideation is heritable via depression (Brent *et al.* 1996; Lieb *et al.* 2005; Brent, 2010).

A study using a molecular genetic risk score to predict depression, found it also predicted suicidal self-injuries (Maciejewski *et al.* 2017). However molecular genetic studies of suicidality have failed to identify replicable associations showing genome-wide significance (Mirkovic *et al.* 2016).

We aimed (1) to describe the prevalence and associations of active and passive suicidal ideation in a Sri Lankan population; (2) to investigate the patterns of heritability of suicidal ideation in a middle income country, where the cultural context of contemplating suicide is expected to be different from the high-income countries studied to date (Bertolote & Fleischmann, 2002; Sumathipala *et al.* 2004); (3) to examine whether some of the heritability of suicidal ideation is independent of depression and (4) to use a discordant MZ twin design, to identify specific measured environmental risk factors that act independently of genetic influences.

## Methods

The study received approvals from King's College London, the University of Sri Jayewardanepura, and the WHO's Research Ethics Committees.

### Study design

The Colombo Twin And Singleton Study (CoTASS) is a population-based twin study with a comparable non-twin sample. Full details of the design of the study are described elsewhere (Siribaddana *et al.* 2008). Briefly, the study took place in the Colombo District of Sri Lanka, a mixed urban and rural area with a population of 2.2 million, which includes the island's capital.

Twins were identified by adding a question to the update of the annual census, asking whether the householder knew of any twins or triplets. We identified 19 302 individuals, of whom 4387 were randomly selected for the present study with a participation rate of 91.7% (*n* = 4024). The present analyses use 3906 twin individuals, including 830 complete MZ pairs and 1110 complete DZ pairs (the remainder being unpaired twins). We conducted a parallel study of non-twins, randomly sampled from the same local areas from which twins were recruited. 2311 non-twins were selected and eligible to participate, of whom 2016 (87.2%) participated. The twin and non-twin samples had similar sex profiles, although the twins (mean age 34.0 years) were younger than the singletons (mean age 43.5 years) (Siribaddana *et al.* 2008). We included all consenting individuals aged 15 years or older who spoke sufficient Sinhala to understand the interview. Individuals were excluded if they failed a mini mental state examination, or where interviews were conducted via a proxy. Interviews took place between 2006 and 2007.

Trained lay researchers visited the participants’ homes and interviewed each twin separately. Interviews and questionnaires were translated at least twice independently in a culturally sensitive manner, then formally reviewed and trialled (Siribaddana *et al.* 2008).

### Measures

Assessment of lifetime-ever suicidal ideation included two items indexing passive ideation (‘have you felt there is no point in living anymore’; ‘have you felt that dying is better than living’) and one indexing active ideation (‘have you thought of committing suicide’). From these items, binary variables were created indicating a positive response to ‘active ideation’, ‘passive ideation’ (without active ideation), or ‘any suicidal ideation’.

We used the Composite International Diagnostic Interview (CIDI) (Robins *et al.* 1988), because it is a diagnostic interview for use by lay-interviewers. Our definition of lifetime-ever depression was slightly less stringent than conventional DSM-IV criteria for a depressive episode (APA, 2000): we ignored the bereavement criterion and the opt-out due to mixed states. We also disregarded the requirement for functional impairment, because it is considered less valid in this population, and the characteristics of the phenotype were very similar with or without this requirement (Ball *et al.* 2010*b*). The CIDI was also used to index tobacco and alcohol use and abuse. Lifetime ever tobacco use was measured as daily use for 1 month or more of cigarettes, cigars, a pipe, snuff or chewed tobacco. Lifetime consumption of at least 12 alcoholic drinks was considered alcohol use. Alcohol abuse was defined as a maladaptive pattern of drinking leading to clinically significant impairment, manifested by one or more of the following: failure to fulfil major role obligations at work/school/home; use when a physical hazard; use-related legal problems; use despite social/interpersonal problems due to alcohol.

The Chalder Fatigue Questionnaire (Chalder *et al.* 1993) was also administered. ‘Abnormal fatigue’ was defined as having at least three of 11 symptoms present at least ‘more than usual’ over the past month (there were no medical exclusions).

Life events were assessed using the List of Threatening Experiences (Brief Life Events Questionnaire) (Brugha & Cragg, 1990) over the past 12 months. The Childhood Experience of Care and Abuse Questionnaire (CECA-Q) (Bifulco *et al.* 2005) was used to assess experience of death or separation from either parent prior to age 17 years.

A further questionnaire based on the Sri Lankan census assessed socio-demographic characteristics and current living environment. Items probed a wide spectrum of household characteristics rather than just detecting the poorest end of the distribution, and these were summed to create a composite variable. We used a binary summary of this composite, indicating those in the top 3/5 of the distribution *v*. the bottom 2/5, because the association with depression was non-linear (Ball *et al.* 2010*b*). This questionnaire also asked about the number of months worked in the previous 12 months, marital status, urban residence and years of education.

### Statistical analysis

#### Epidemiological analyses (twins and non-twins)

A database combining the twin and non-twin data were constructed in SPSS-10. Analyses were performed in Stata version 9.2 (Stata-Corp, College Station, Texas). Analyses were corrected for the non-independence of twins within pairs, using the *Huber–White–Sandwich* (*robust*) *estimator of variance* (Williams, *2000*).

Multiple logistic regression analyses were used to identify socio-demographic factors that were independently associated with suicidal ideation. These factors were then controlled for in subsequent logistic regression models, which examined the association with social circumstances, psychiatric symptoms and behaviours.

#### Genetic analyses of ‘any suicidal ideation’ (twins only)

We used standard twin modelling as implemented in Mx for Windows (www.vcu.edu/mx/index.html) to estimate the relative contributions of additive genetics (A), shared environments (C) and non-shared environments (E) to suicidal ideation. This is done by examining the similarity of monozygotic (MZ) pairs of twins, and comparing this with the similarity of dizygotic (DZ) pairs of twins. For example, higher correlations within MZ compared with DZ pairs indicates A, because shared genes are the only explanation for greater similarity. C is indicated by any environmental exposure that makes twins within a pair (both MZ and DZ) similar to one another (e.g. family-wide poverty). E is indicated by any environmental factor that makes one twin different from the co-twin (e.g. an accident that affects only one twin within a pair), and so E is calculated as the amount of dissimilarity within MZ pairs (note E also incorporates measurement error). Latent ACE variable parameter constraints were applied, then standardized. It is possible to calculate a parameter indexing dominance genetic variance (D) instead of C; this is indicated if the correlation for MZ pairs is greater than double that for DZ pairs.

We examined ‘any suicidal ideation’ (rather than active or passive separately) because there were only a small number of ‘concordant affected’ pairs (those in which both twins reported ideation). This also limited our ability to generate latent ACE variable parameter models to univariate only; bivariate analyses were performed using logistic regression models (see c and d below).

The estimated genetic model is compared with the observed data in order to produce the maximum likelihood fit of the model. This genetic model fit is compared with that of a fully saturated model of the correlations. Models that are nested within one another can be compared using a chi-squared test.

Tetrachoric correlations were used because the data are binary. This method assumes that liability to suicidal ideation is normally distributed throughout the population, with affected individuals having exceeded a certain threshold of liability. Liability thresholds were estimated separately for men and women. The effect of age was accounted for through regression coefficients on the liability thresholds, as has been described previously (Reynolds *et al.* 2006).

#### Genetic analyses of the relationship between ‘any suicidal ideation’ and depression (twins only)

Logistic regression models were run, which predicted ‘any suicidal ideation’ in one twin (‘proband’) from the same variable in the co-twin. This indicates familial contributions to suicidal ideation. Next, the models were re-run while controlling for depression in the co-twin, to indicate whether the familial influences on suicidal ideation are independent of familial influences on depression. The age control for these analyses was linear.

Finally, the results were compared across zygosity groups. A significant interaction according to zygosity (i.e. a stronger association in MZs than DZs) indicates the existence of a genetic contribution to suicidal ideation, which is independent of depression.

#### MZ differences models: ‘true’ environmental associations

The social circumstances identified to correlate with suicidal ideation [in the epidemiological analysis described in (*a*)] might be construed as discrete insults that impact on an individual, which then cause the outcome (suicidal ideation). An alternative explanation is that these social circumstances are markers of associated genetic tendencies (A) or wider aspects of family upbringing (C). If however, a correlation remains after we have ruled out the effects of A and C, there is more chance it is involved in a truly environmental causal pathway. By looking at the differences within MZ pairs of twins, it is possible to confirm whether a social circumstance is associated with suicidal ideation via non-shared environments (E). Therefore, we measured the difference in suicidal ideation within each pair of MZ twins, and separately measured the difference in the social circumstances within each pair of MZ twins. Ordered logistic regression models were used to examine the association between the difference in suicidal ideation within each MZ twin pair, with the difference in social circumstances within each MZ twin pair.

The twin analyses described in (c) and (d) were only controlled for age, sex and ethnicity, on the basis that these factors are determined at birth, so are temporally prior and could not be an ‘outcome’ of suicidal ideation or depression.

## Results

Data are presented for 5922 participants. Reported lifetime ever suicidal ideation was 13.0% (11.7–14.3) for men and 21.8% (20.3–23.2) for women. Overall 6.2% (CI 5.6–6.9) had experienced active ideation, and 11.5% (CI 10.7–12.4) reported passive ideation only (Table 1). Adjusting only for age, owing to the difference in mean age between twins and non-twins in the study, there was no significant difference in prevalence of any suicidal ideation (twins 16.9%, CI 15.6–18.2, non-twins 19.4%, CI 17.7–21.1, *p* = 0.51).

**Table 1.** Socio-demographic factors associated with suicidal ideation

	Any suicidal ideation *n*/*N* (%)	Any suicidal ideation: multiple logistic regression OR (95% CI)	Active *n*/*N* (%)	Active: multiple logistic regression OR (95% CI)	Passive *n*/*N* (%)	Passive: multiple logistic regression OR (95% CI)
All	1051/5922 (17.8)	**–**	368/5921 (6.2)	**–**	682/5922 (11.5)	**–**
Sex						
Male	354/2720 (13.0)	1 [Ref]	113/2720 (4.2)	1 [Ref]	241/2720 (8.9)	1 [Ref]
Female	697/3202 (21.8)	**1.73 (1.50–2.01)**	255/3201 (8.0)	**1.84 (1.45–2.34)**	441/3202 (13.8)	**1.55 (1.29–1.85)**
Twin status						
Singleton	392/2016 (19.4)	1 [Ref]	123/2016 (6.1)	1 [Ref]	269/2016 (13.3)	1 [Ref]
Twin	659/3906 (16.9)	0.96 (0.82–1.12)	245/3905 (6.3)	1.09 (0.85–1.39)	413/3906 (10.6)	0.90 (0.75–1.08)
Age category						
15–29y	315/2199 (14.3)	1 [Ref]	134/2198 (6.1)	1 [Ref]	180/2199 (8.19)	1 [Ref]
30–44y	347/1907 (18.2)	1.16 (0.96–1.39)	131/1907 (6.9)	0.98 (0.75–1.28)	216/1907 (11.3)	**1.27. (1.01–1.60)**
45y+	389/1816 (21.4)	1.13 (0.93–1.37)	103/18 116 (5.7)	**0.59 (0.43–0.82)**	286/1816 (15.8)	**1.57 (1.25–1.97)**
Ethnicity						
Sinhala	959/5465 (17.6)	1 [Ref]	344/5464 (6.3)	1 [Ref]	614/5465 (11.2)	1 [Ref]
Tamil	42/165 (25.5)	1.43 (0.93–2.19)	14/165 (8.5)	1.17 (0.61–2.23)	28/165 (17.0)	1.49 (0.89–2.50)
Muslim	49/272 (18.0)	0.87 (0.60–1.27)	10/272 (3.7)	**0.46 (0.22–0.98)**	39/272 (14.3)	1.18 (0.78–1.79)
Other	1/20 (5.00)	0.21 (0.03–1.72)	0/20 (0.0)	NA	1/20 (5.00)	0.38 (0.05–3.12)
Marital status						
Never married	304/2006 (15.2)	1 [Ref]	113/2005 (5.6)	1 [Ref]	190/2006 (9.47)	1 [Ref]
Married	602/3543 (17.0)		202/3543 (5.7)		400/3543 (11.3)	
Previously married^a^	145/372 (39.0)	**2.18 (1.69–2.80)**	53/372 (14.3)	**2.64 (1.79–3.89)**	92/372 (24.7)	**1.62 (1.22–2.16)**
Years at school						
11+	511/3724 (13.7)	1 [Ref]	192/3723 (5.2)	1 [Ref]	318/3724 (8.5)	1 [Ref]
Up to 10	531/2156 (24.6)	**1.84 (1.59–2.13)**	174/2156 (8.1)	**1.63 (1.29–2.06)**	357/2156 (16.6)	**1.80 (1.52–2.13)**
Residence						
Semi-urban	573/3603 (15.9)	1 [Ref]	213/3601 (5.9)	1 [Ref]	359/3603 (10.0)	1 [Ref]
Urban	478/2320 (20.6)	**1.29 (1.11–1.51)**	155/2320 (6.7)	1.16 (0.92–1.48)	323/2320 (13.9)	**1.32 (1.10–1.57)**

a Comparing previously married (widowed OR separated OR divorced), with combined group of never married OR married.

Where *n* differs by 1 or 2 within a row, this is due to missing data. Bold values indicate *p* < 0.05.

### Socio-demographic factors

Suicidal ideation was consistently associated with female sex, being widowed, separated or divorced and low educational attainment (Table 1). Active ideation was most common amongst 30–44 year olds, whereas passive ideation became more prevalent with increasing age. Ethnicity was not associated with ‘any suicidal ideation’, but being Muslim was a protective factor with regard to active ideation. Urban living was associated with passive ideation.

### Social circumstances

Parental loss as a child, one or more life events, under-employment (working 2–10 months in the previous 12 months) and having a low standard of living were uniformly associated with all types of suicidal ideation (Table 2).

**Table 2.** Environmental exposures, psychiatric symptoms and behaviours associated with suicidal ideation

	Any suicidal ideation *n*/*N* (%)	Any suicidal ideation, multiple logistic regression OR (95% CI)	Active *n*/*N* (%)	Active: multiple logistic regression OR (95% CI)	Passive *n*/*N* (%)	Passive: multiple logistic regression OR (95% CI)
*Environmental exposure models*						
Months worked in past 12 months^a^						
11–12	373/2452 (15.2)	1 [Ref]	135/2452 (5.5)	1 [Ref]	238/2452 (9.7)	1 [Ref]
2–10	158/656 (24.1)	**1.53 (1.22–1.91)**	66/656 (10.1)	**1.62 (1.17–2.24)**	92/656 (14.0)	**1.32 (1.00–1.75)**
0–1	498/2588 (19.2)	0.96 (0.81–1.13)	163/2587 (6.3)	0.81 (0.62–1.06)	334/2588 (12.9)	1.06 (0.87–1.30)
Parents: died/separated from them before 17yrs^a^						
No	678/4327 (15.7)	1 [Ref]	219/4326 (5.1)	1 [Ref]	458/4327 (10.6)	1 [Ref]
Yes	370/1576 (23.5)	**1.56 (1.34–1.82)**	147/1576 (9.3)	**1.86 (1.48–2.34)**	223/1576 (14.2)	**1·29 (1·08–1·55)**
Life events in preceding yr^a^						
None	255/2517 (10.1)	1 [Ref]	64/2517 (2.5)	1 [Ref]	191/2517 (7.6)	1 [Ref]
1+	790/3388 (23.3)	**2.61 (2.22–3.06)**	300/3387 (8.9)	**3.53 (2.68–4.67)**	489/3388 (14.4)	**1.96 (1.63–2.36)**
Standard of living^a^						
Top 3/5	475/3564 (13.3)	1 [Ref]	155/3563 (4.4)	1 [Ref]	319/3564 (8.95)	1 [Ref]
Bottom 2/5	576/2358 (24.4)	**1.78 (1.53–2.07)**	213/2358 (9.0)	**1.97 (1.57–2.48)**	363/2358 (15.4)	**1.52 (1.28–1.82)**
*Psychiatric symptom and behaviour models*						
Depression^b^						
Absent	744/5261 (14.1)	1 [Ref]	211/5260 (4.0)	1 [Ref]	532/5261 (10.1)	1 [Ref]
Present	307/661 (46.4)	**3.75 (3.09–4.55)**	157/661 (23.8)	**5.32 (4.12–6.88)**	150/661 (22.7)	**1.82 (1.44–2.30)**
Abnormal fatigue^b^						
Absent	624/4762 (13.1)	1 [Ref]	214/4761 (4.5)	1 [Ref]	409/4762 (8.6)	1 [Ref]
Present	423/1147 (36.9)	**2.55 (2.16–3.02)**	153/1147 (13.3)	**2.05 (1.58–2.65)**	270/1147 (23.5)	**2.27 (1.88–2.75)**
Alcohol abuse^b^						
No use	745/4068 (18.3) Men only: 83/985 (8.4)	1 [Ref]	266/4067 (6.5) Men only: 25/985 (2.5)	1 [Ref]	478/4068 (11.8) Men only: 58/985 (5.9)	1 [Ref]
Use but not abuse	229/1603 (14.3) Men only: 194/1484 (13.1)	1.16 (0.91–1.48)	70/1603 (4.4) Men only: 56/1484 (3.8)	1.08 (0.73–1.59)	159/1603 (9.9) Men only: 138/1484 (9.3)	1.19 (0.90–1.57)
Abuse	76/249 (30.5) Men only: 76/249 (30.5)	**2.29 (1.55–3.39)**	31/249 (12.5) Men only: 31/249 (12.5)	**2.15 (1.23–3.76)**	45/249 (18.1) Men only: 45/249 (18.1)	**1.97 (1.24–3.14)**
Tobacco use^b^						
Absent	827/4813 (17.2)	1 [Ref]	286/4812 (5.9)	1 [Ref]	540/4813 (11.2)	1 [Ref]
Present	223/1108 (20.1)	**1.45 (1.13–1.85)**	81/1108 (7.3)	**2.01 (1.37–2.95)**	142/1108 (12.8)	1.14 (0.87–1.50)

Bold values indicate *p* < 0.05.

a Environmental exposure models controlled for socio-demographic factors: sex, age, ethnicity, being previously married, years of schooling and urban residence.

b Psychiatric symptoms and behaviour models controlled for the above socio-demographic factors and mutually controlled for the measures of depression, abnormal fatigue, alcohol use and abuse and tobacco use.

### Psychiatric symptoms and behaviours

There were robust associations between all types of suicidal ideation and depression, abnormal fatigue and alcohol abuse (Table 2). Depression was more strongly associated with active than passive ideation.

Alcohol use (excluding alcohol abuse) was not associated with suicidal ideation; whereas alcohol abuse was associated with all types of suicidal ideation. Tobacco use was associated with active but not passive suicidal ideation. Alcohol abuse was identified solely in males: 9.2% of men abused alcohol. A very low proportion (3.7%) of women used alcohol compared with 63.1% of men, and only 2.0% of women used tobacco compared with 38.4% of men.

### Twin correlations and genetic models

Twin tetrachoric correlations were calculated in Mx, controlling for age. One linear age parameter was used for each sex. The age parameter was statistically significant so was retained for the purposes of calculating ACE parameters. The thresholds (which indicate prevalence) could not be equated across sex (due to higher prevalence in women than men), but thresholds could be equated across zygosity within men and women, without significant reduction in fit (indicating that the prevalence did not differ across zygosity groups).

The tetrachoric correlations in women (MZ: 0.53; DZ: 0.40) suggest that a mixture of additive genetic and common environmental factors contribute to familial similarity in suicidal ideation. The tetrachoric correlations in men (MZ: 0.63; DZ: 0.10) suggest a dominance genetic pattern, since the figure for MZs is more than double that for DZs (however, it should be noted that there were only four DZ concordant affected male pairs, making the tetrachoric correlation estimate imprecise). We therefore tested model fit for both ACE and ADE in men (Table 3), and both were an adequate fit. However, the subsequent models showed that it is possible to drop the C or D parameters and this is reflected in Table 4 where the confidence intervals for the C and D parameters include zero. Therefore the correlations were adequately modelled with only A and E parameters (Model 7, Tables 3 & 4: change in -2 log likelihood = 0.724, df = 2, *p* = 0.696, change in AIC −3.276). Furthermore, the estimated values of A and E could be equated across sex without significant loss of fit (Model 8, Tables 3 & 4: change in −2 log likelihood 0.930, df = 3, *p* = 0.818, change in AIC −5.070). Therefore, the variance in liability to suicidal ideation, in both men and women, was influenced by additive genetic factors 57% (95% CI 47–66) and non-shared environmental factors 43% (95% CI 34–53).

**Table 3.** Genetic model fit statistics for ‘any suicidal ideation’

Model	−2 log likelihood	Degrees of freedom (df)	Compare to model	Change in −2 log likelihood	Change in df	*p*	Change in AIC
1. Saturated	3300.893	3827	–	–	–	–	–
2. ACE_ACE	3302.854	3828	1	1.961	1	0.16	−0.039
3. ADE_ACE	3303.414	3828	1	2.521	1	0.112	0.521
4. AE_ACE	3303.430	3829	3	0.016	1	0.900	−1.984
5. ADE_AE	3303.970	3829	3	0.556	1	0.456	−1.444
6. ADE_CE	3314.878	3829	3	11.464	1	0.001	9.464
7. AE_AE	3304.138	3830	3	0.724	2	0.696	−3.276
8. AE (equated across sex)	3304.344	3831	3	0.930	3	0.818	−5.070

AIC, Akaike's information criterion.

*Note 1: Because the correlations reported above suggested dominance genetics in men, so an ADE model was attempted in men as well as the more standard ACE model.*

*Note 2: male model written first, eg ADE_ACE means a model using ADE in men and ACE in women.*

**Table 4.** Percentage of variance in ‘any suicidal ideation’ explained by ACDE

	Male parameters			Female parameters		
Model	A	C or D	E	A	C	E
2. ACE_ACE	57 (21–76)	3 (0–30)	40 (24–61)	30 (0–65)	24 (0–55)	47 (35–60)
3. ADE_ACE	57 (10–77)	4 (0–57)	38 (23–58)	41 (11–66)	13 (0–42)	45 (34–58)
7. AE_AE	60 (41–76)	–	40 (24–59)	55 (43–67)	–	45 (33–57)
8. AE (equated across sex)	57 (47–66)	–	43 (34–53)	57 (47–66)	–	43 (34–53)

Note: only standardized estimates of variance are presented.

The tetrachoric correlation for DZ opposite sex pairs was 0.31. This is mid-way between the figure for all-male and all-female DZ pairs, suggesting that sex differences are quantitative rather than qualitative (i.e. the same individual aetiological factors affect men and women, but to different degrees). We were able to calculate the tetrachoric correlations separately for active and passive ideation among women. These correlations (active ideation MZ 0.52, DZ 0.39; passive ideation MZ 0.46, DZ 0.34) were similar to those for ‘any suicidal ideation’.

### The heritability of suicidal ideation that is independent of depression

Model 1 (Table 5) shows significant association between ideation in the proband (twin 1) with the co-twin (twin 2), showing that familial factors (i.e. genetic, A, or shared-family environmental factors, C), contribute to suicidal ideation. Model 2 shows that there is still a familial contribution to suicidal ideation after controlling for the familial effect of depression. The effect of zygosity is significant in Model 2, showing there is a *genetic* contribution to suicidal ideation that is independent of the heritability of depression. However, when analysed separately by gender, this was statistically significant in men only. This suggests that amongst women, the genetic component of suicidal ideation (Table 4) is largely mediated through depression.

**Table 5.** Cross-twin logistic regression to examine the heritability of ideation independent of the heritability of depression

	Predicting Twin 1 ideation from Twin 2 ideation						
	1. Controlling for age, sex and ethnicity OR (95% CI)	2. As Model 1, plus controlling for Twin 2 depression OR (95% CI)	3. As Model 1, plus controlling for Twin 2 alcohol abuse OR (95% CI)	4. As Model 1, plus controlling for Twin 2 depression and alcohol abuse OR (95% CI)	Effect of zygosity in Model 2	Effect of zygosity in Model 3	Effect of zygosity in Model 4
All	3.80 (2.90–4.99)	3.52 (2.67–4.64)	3.73 (2.88–4.97)	3.51 (2.65–4.63)	*Z* = 2.80, *p* = 0.005	*Z* = 2.66, *p* = 0.008	*Z* = 2.73, *p* = 0.006
MZ	5.68 (3.72–8.69)	5.24 (3.41–8.05)	5.63 (3.68–8.61)	5.20 (3.39–7.99)			
DZ (incl DZOS)	2.71 (1.88–3.91)	2.51 (1.72–3.65)	2.68 (1.86–3.87)	2.49 (1.71–3.62)			
							
*Men* (*excl DZOS*)	4.19 (2.26–7.79)	3.84 (2.02–7.31)	4.06 (2.17–7.59)	3.76 (1.96–7.19)	*Z* = 2.58, *p* = 0.01	*Z* = 2.46, *p* = 0.014	*Z* = 2.57, *p* = 0.010
MZM	8.07 (3.59–18.11)	7.17 (3.16–16.29)	7.86 (3.48–17.74)	7.09 (3.10–16.23)			
DZM	1.34 (0.42–4.27)	1.27 (0.38–4.22)	1.24 (0.37–4.11)	1.19 (0.35–4.09)			
							
*Women* (*excl DZOS*)	4.22 (2.89–6.18)	3.94 (2.68–5.81)	4.22 (2.89–6.18)	3.94 (2.68–5.81)	*Z* = 1.09, *p* = 0.28	*Z* = 1.04, *p* = 0.299	*Z* = 1.09, *p* = 0.277
MZF	4.96 (3.04–8.08)	4.64 (2.84–7.57)	4.96 (3.05–8.08)	4.64 (2.84–7.57)			
DZF	3.16 (1.70–5.88)	2.95 (1.55–5.62)	3.16 (1.70–5.88)	2.95 (1.55–5.62)			
							
*DZ OS*	2.74 (1.63–4.60)	2.54 (1.50–4.30)					

DZOS, DZ opposite sex pairs.

Models 3 and 4 have been included for women for completeness; though as we note earlier, alcohol abuse is very rare in women in this population so these models are largely unchanged after accounting for female alcohol abuse.

Models 3 and 4 of Table 5 show that, in men, the heritable contribution to suicidal ideation is independent from any heritable contribution to alcohol abuse, as well as being independent from any heritable contribution to depression.

### Socio-demographic factors associated with suicidal ideation, independent of genetic influences

Several of the socio-demographic factors studied were still associated with suicidal ideation when examined within pairs of MZ twins, indicating the causal pathway is operating via non-shared environmental factors, E, rather than being confounded by genes or the wider family environment (Table 6). These included living without a marital partner, stressful life events and standard of living. For years at school, urban residence and level of employment in the past year, the strength of association with suicidal ideation diminished when looking at differences within MZ pairs; i.e. these associations were no longer statistically significant when familial effects (A and C) had been controlled for.

**Table 6.** Socio-demographic and environmental associations independent of genes *(*monozygotic twin differences*)*

	Number of pairs discordant for the environmental variable	OR (95% CI) for the association between within-pair difference in (i) suicidal ideation and (ii) social/ demographic factors		
Socio-demographic/ Environmental factor		All	Men	Women
Widowed/ Separated/Divorced	46	**3.76** (**1.94–7.30)**	**10.19** (**2.44–42.62)**	**2.59** (**1.26–5.36)**
Years at school (⩾/>10)	87	1.18 (0.69–2.02)	1.25 (0.50–3.11)	1.17 (0.59–2.32)
Urbanisation	36	0.95 (0.40–2.25)	2.08 (0.35–12.36)	0.77 (0.31–1.94)
Underemployed (2–10mths work in past 12 mths)	118	1.35 (0.85–2.14)	0.87 (0.37–2.03)	1.63 (0.94–2.84)
Unemployed (0–1mths)	174	0.97 (0.66–1.43)	0.47 (0.18–1.25)	1.10 (0.74–1.62)
Life event^a^	390	**1.50 (1.22–1.84)**	1.43 (0.99–2.06)	**1.54 (1.20–1·97)**
Standard of living (binary, lower 2/5^th^ *v*. upper 3/5th)	169	**1.63 (1.10–2.40)**	**2.36 (1.18–4.75)**	1.40 (0.88–2.23)

Based on *N* = 824 pairs of MZ twins; of whom 151 pairs (18.3%) were discordant for suicidal ideation.

Note no significant sex interaction terms were found. Bold values indicate *p* < 0.05.

a Life events were categorized into 0, 1, or 2 or more events per person.

## Discussion

We aimed to examine the prevalence and risk factors for suicidal ideation in a population-representative sample from an LMIC and to investigate the relative contribution of genetic and environmental influences on suicidal ideation. Key findings were that 13% of male twins and singletons and almost 22% of females reported suicidal ideation in their lifetime, and this was mainly passive ideation (12%) rather than active ideation, which had been experienced by 6% [similar to the 7.3% prevalence reported for Colombo in the SUPRE-MISS study (Bertolote *et al.* 2005), but lower than the 9.2% average reported in the World Mental Health survey, where country prevalence ranged from 3.0 to 15.9% (Nock *et al.* 2008)].

There was no difference in the prevalence of suicidal ideation for singletons or twins or across zygosity groups. This is important, because previous literature suggests a modest protective effect of being a twin on completed suicides (Tomassini *et al.* 2003), which would undermine the generalizability of twin findings regarding suicidal ideation to the wider population.

### Suicidal ideation as a familial trait

We showed higher concordance of suicidal ideation among MZ than DZ twins, consistent with a genetic influence on suicidal ideation. This translated to a significant contribution of additive genetic factors (heritability estimate: 57%) for suicidal ideation. Non-shared environmental effects also contributed substantially to the risk of suicidal ideation (43%) whereas the effect of shared family environment did not.

Twin studies of completed suicide and attempted suicide in Europe and the USA (Roy *et al.* 1991; Roy *et al.* 1995; Roy & Segal, 2001) have shown similar effects. Studies of suicidal ideation are rare. An Australian twin study (Statham *et al.* 1998) estimated a similar genetic contribution (45%) to the variance in suicidal thoughts and behaviours, with a higher estimate of heritability (55%) obtained when serious suicide attempts were considered. After controlling for socio-demographic, personality, psychiatric, traumatic event and family history variables, a history of suicide attempt or persistent suicidal thoughts in a co-twin remained a significant predictor of suicidal thoughts and behaviour in MZ twins, but not in DZ twin pairs, implying some genetic contribution to suicidal thoughts or behaviours independent of clinical and social covariates.

In twin men from the US Vietnam Era Twin Registry, suicidal ideation was found to be influenced by additive genetic (47%) and non-shared environmental (53%) effects (Fu *et al.* 2002). This study also showed some of the heritability of suicidal ideation was independent of psychiatric disorders with 36% additive genetic and 64% non-shared environmental contributions remaining after adjustment (Fu *et al.* 2002).

Analysing published data from Roy *et al*. (1991), McGuffin *et al.* (2001) estimated heritability for completed suicide to be 43% (25–60%). This paper also suggested that the overlap between genes predisposing to affective disorders and those related to suicidality is unlikely to be complete, as only about half those completing suicide had a diagnosis of depression.

We found that in women the genetic component appeared to be largely mediated through depression; but in men there was a significant genetic effect independent of depression and alcohol abuse. In men, other unmeasured forms of psychopathology (e.g. externalizing traits), which are genetically driven, may make a contribution to suicidal ideation. Our study demonstrates a heritable component of suicidal ideation, which is incompletely explained by measured psychopathology, suggesting a potential direct heritable effect on suicidal behaviour. This emphasizes the clinical importance of family history of suicidal behaviours, even in patients without any diagnosed psychiatric disorder. However, there is a need for further studies to assess the aetiological overlap between suicidal behaviours and a range of other psychiatric disorders, for example a recent register study has shown that patients with Chronic Fatigue Syndrome have a higher mortality from suicide than the general population (Roberts *et al.* 2016).

### Environmental associations with suicidal ideation

Examining the differences within MZ pairs of twins allowed us for the first time to identify environmental associations of suicidal ideation that are independent of genetic factors. Loss of marital relationships in both sexes, stressful life events in women and lower standard of living in men were associated with suicidal ideation independent of genetic effects. This means these are true environmental effects rather than markers of inheritance or shared upbringing. These factors are typically found to be correlated with depression, but previous analyses in this sample found the only associations of depression that were independent of genetic effects were in men not women: low standard of living, leaving school at a young age and stressful life events (Ball *et al.* 2010*a*). This suggests different aetiological pathways leading to suicidal ideation compared with depression.

For education (years at school), urbanization and level of employment in the last year, there was no such environmentally-mediated association with suicidal ideation. This suggests that the latter three environmental variables are confounded – perhaps a genetic factor makes people more likely to experience this environmental variable and to experience suicidal ideation, or perhaps it is a marker of some other aspect of family upbringing that is associated with both the environmental variable and suicidal ideation. For example, genetic factors relating to low IQ might mean an individual leaves school early and moves to a town to look for employment but remains under-employed and independently many studies suggest that low IQ is associated with an elevated risk of suicide (Gunnell *et al.* 2005).

Furthermore, the important cultural factors that account for differences in alcohol (Zavos *et al.* 2015) and tobacco consumption (Zavos *et al.* 2012) amongst males and females means that the association we observed for these variables with suicidal ideation was driven by effects in men, and cannot be generalized to both sexes.

### Limitations

We used a population-based twin sample with exceptionally high participation rates, making our results more generalizable than many previous twin studies based on volunteer registries. However the epidemiological analyses are based on cross-sectional data and therefore causation cannot be inferred. Life-event data were measured over the past-year (whereas suicidal ideation and depression were measured on a lifetime-ever basis), further limiting our ability to ascertain causal links from the epidemiological data.

Despite presenting the largest study of its kind from a LMIC, our power to detect sex differences in ACE models was low and we present models, which equate model parameters between men and women. Our genetic analyses were also limited to ‘any suicidal ideation’ rather than examining active and passive ideation separately. Finally, power was too low to run bivariate ACE models, so instead we used logistic regression models to explore the aetiology of the associations between suicidal ideations on the one hand, and depression, alcohol abuse and socio-demographic or environmental factors on the other.

As suicidal ideation, intentions, plans, behaviours and attempts are on a spectrum it is difficult to find valid and appropriate, as well as culturally-sensitive, screening questions for suicidal ideation that are comparable with other international studies (Samaraweera *et al.* 2010). However this study was preceded by local research to investigate whether inhabitants of a suburb of Colombo would volunteer their life weariness and suicidal ideation (Sumathipala *et al.* 2004) and concluded that individuals would disclose them if asked directly.

## Conclusions

The moderate degree of heritability of suicidal ideation in this twin study in Sri Lanka was similar to estimates made for any suicidal behaviour (ideation or attempts) or completed suicides from other population-based epidemiological twin studies conducted in different parts of the world. Twin/non-twin status was associated with neither passive nor active suicidal ideation, supporting generalizability of the findings to the general population. In addition to deepening the understanding of heritable components to suicidal ideation, what is apparent is that social adversity (life events, marital breakup and socio-economic disadvantage) are important environmental risk factors. which act independently from genetic risk.
